# Hydroxylated vs. Carboxylated Nanotubes: Differential Impacts on Fall Armyworm Development, Reproduction, and Population Dynamics

**DOI:** 10.3390/insects16080748

**Published:** 2025-07-22

**Authors:** Zhao Wang, Syed Husne Mobarak, Fa-Xu Lu, Jing Ai, Xie-Yuan Bai, Lei Wu, Shao-Zhao Qin, Chao-Xing Hu

**Affiliations:** 1 College of Life and Health Science, Kaili University, 3 Kaiyuan Road, Kaili 556011, China; 2 Guizhou Key Laboratory of Agricultural Biosecurity, Institute of Entomology, Institute of Plant Health and Medicine, Guizhou University, Guiyang 550025, China

**Keywords:** *Spodoptera frugiperda*, MWCNTs-OH, MWCNTs-COOH, developmental inhibition, reproductive disruption, population dynamics

## Abstract

Carbon nanotubes are promising in agriculture for improving crop resilience and delivering agrochemicals. However, their effects on insect pests, especially chewing pests such as the fall armyworm (*Spodoptera frugiperda*), remain underexplored. In this study, we investigated how two types of functionalized multi-walled carbon nanotubes—hydroxylated (MWCNTs-OH) and carboxylated (MWCNTs-COOH), both obtained from Jiangsu Xianfeng Nano (Nanjing, China)—affect the pest’s development and reproduction. Using an age-stage two-sex life table approach, we fed larvae diets containing 0.04, 0.4, or 4 mg/g of these nanomaterials. Both types of MWCNTs exhibited concentration-dependent inhibitory effects. At the highest dose (4 mg/g), larval development was significantly prolonged, adult pre-oviposition periods increased, and fecundity (egg production) sharply declined, especially with MWCNTs-OH. Population growth parameters were also suppressed: net reproductive rate (*R*_0_), intrinsic rate of increase (*r*), and finite rate of increase (*λ*) were reduced at 4 mg/g, particularly with MWCNTs-OH, while mean generation time (*T*) was extended with MWCNTs-COOH. Overall, MWCNTs-OH demonstrated a greater inhibitory impact compared to MWCNTs-COOH. These findings suggest that functionalized MWCNTs could serve as potential novel pest control agents against *S. frugiperda* by impeding its development and reproduction.

## Simple Summary

The fall armyworm is a destructive caterpillar that attacks many crops worldwide and is difficult to control. Our study explored a new method to deal with this pest using tiny carbon tubes known as carbon nanotubes. We added two types of these nanotubes (with slightly different properties) into the caterpillars’ food at various doses. At higher doses, these nanomaterials noticeably slowed the insects’ growth and greatly reduced their ability to reproduce, especially with one type of nanotube. In other words, the caterpillars took longer to develop into moths, and the adult moths laid far fewer eggs when the nanotubes were present. This finding is important because it points to a potential new pest control strategy. Such carbon-based nanomaterials could serve as an innovative, environmentally friendly tool for farmers to protect crops against the fall armyworm, potentially reducing the need for traditional chemical pesticides.

## 1. Introduction

The fall armyworm (*Spodoptera frugiperda*) is a destructive pest threatening crops worldwide. Native to the Americas, it spread to Africa in 2016 and Asia in 2018, subsequently affecting over one million hectares of corn in China [1]. This pest attacks 186 plant species, causing yield losses of up to 58% [2]. Its rapid reproduction, adaptability, and increasing resistance to pesticides exacerbate its agricultural impact [3]. In Africa, corn yields have dropped by 25–67%, and in China, potential losses could reach CNY 328.35 billion (approximately USD 46 billion) [4]. Current methods for controlling *S. frugiperda* include field management, biological control, and chemical pesticides; however, pesticide resistance remains a significant challenge. This underscores the urgent need for safe, efficient, and environmentally friendly approaches to manage this destructive pest.

In the realm of crop pest control, novel insecticides that utilize nanomaterial carriers have emerged as a promising approach. Research by Jiang et al. [5] demonstrated that these nanopesticides significantly enhance insecticidal effectiveness and prolong efficacy against pests. This advancement presents a unique opportunity to improve pesticide efficiency and combat pest resistance, underscoring the importance of integrating nanotechnology into pest management strategies [6]. Among various nanomaterials, carbon nanomaterials (CNMs) stand out due to their exceptional properties. In particular, carbon nanotubes (CNTs) have garnered attention in agricultural production for their unique stiffness, strength, elasticity, and superior performance compared to other fibrous nanomaterials [7]. Interest in CNTs in agriculture has surged over the past two decades. Chowdhury et al. [8] reported that 40% of their applications serve as additives and active ingredients. The versatility of CNMs extends beyond pest control, as they can penetrate plant cells and modify physiological and morphological characteristics, enhancing growth. Sakthivel et al. [9] noted that CNMs are even used in innovative fertilizers to boost plant productivity. Furthermore, due to their strong penetration capability, CNMs can function as smart delivery systems for agricultural chemicals, organic molecules, and even transport DNA molecules or oligonucleotides into plant cells [7]. Recent studies have demonstrated that multi-walled CNTs play a crucial role in sustainable pesticide applications by converting pesticides into nanoparticles that are gradually released through the nanotube walls [9]. However, the ecological risks associated with deploying CNTs in agroecosystems also warrant careful consideration. Carbon nanotubes are extremely stable, may persist in soil and water, and studies have shown that they can accumulate in plants and potentially enter the food chain [10]. Thus, the benefits of CNT-based pest control must be carefully weighed against potential long-term environmental impacts, and thorough assessments of persistence, degradability, and non-target effects are required.

Currently, our understanding of the toxicity of nanomaterials in agricultural systems is still developing due to limited data and detailed studies on the toxicity of CNTs. Reports suggest that certain nanomaterials do not negatively affect beneficial insects within agricultural ecosystems. For instance, natural carbon-based quantum dots (NCDs) were found to be non-toxic, causing no adverse effects on the hemocytes of the silkworm (*Bombyx mori*) [11]. However, research on CNTs in insects remains relatively limited and typically focuses on specific species and traits, highlighting the need for additional studies on the application of nanomaterials in pest control. Investigating key biological parameters—such as survival rate, fecundity, longevity, and intrinsic growth rate of insect pests—provides a robust framework for predicting pest control outcomes [12].

To comprehensively assess the impact of hydroxylated (-OH) and carboxylated (-COOH) multi-walled carbon nanotubes (MWCNTs-OH and MWCNTs-COOH) on *S. frugiperda* populations, this study employed the age-stage two-sex life table approach. This method is widely recognized as a valuable tool for investigating insect reproduction, development, and population dynamics [13,14]. The selection of MWCNTs was based on their unique tubular structure, which provides a high surface area and loading capacity for agrochemical delivery [15]. Additionally, functionalized MWCNTs, such as MWCNTs-OH and MWCNTs-COOH, were chosen because their surface modifications enhance dispersibility, biocompatibility, and the ability to efficiently bind and transport active molecules compared to unmodified nanotubes [16,17].

The findings of this study provide a preliminary basis for considering MWCNTs-OH and MWCNTs-COOH as novel tools for managing *S. frugiperda*. Our results demonstrate the potential of these nanomaterials to suppress fall armyworm populations while also underscoring the necessity of further evaluations regarding their safety and field applicability.

## 2. Materials and Methods

### 2.1. Insect Mass Culture

Adult *S. frugiperda*, both males and females, were collected from a cornfield in Guiyang, Guizhou, China (26°43′ N, 106°67′ E) in August 2020 using a light trap. They were subsequently reared in a growth chamber at 25 ± 1 °C, 65 ± 5% relative humidity, and a 14L:10D photoperiod at the Institute of Entomology, Guizhou University. Adults were provided with a 10% honey solution, and moistened filter papers were offered as oviposition substrates inside rearing cages (30 × 30 × 30 cm). To prevent cannibalism, larvae were reared individually on an artificial diet (as described by Li et al. [18]) in culture plates: 24-well plates for the 1st and 2nd instars, 12-well plates for the 3rd and 4th instars, and 6-well plates for the 5th and 6th instars. All bioassays for this study were conducted in 2023, during the fourth consecutive year of laboratory colony rearing. To prevent genetic degeneration and maintain colony vigor, field-collected *S. frugiperda* individuals were periodically introduced, and a large breeding population was maintained in each generation.

### 2.2. Preparation of Artificial Diet

The artificial larval diet was prepared following the protocol described by Li et al. [18], with slight modifications. First, 600 mL of deionized water was heated to a boil, after which 8 g of agar-agar was added with continuous stirring until the solution turned white. Simultaneously, another 600 mL of deionized water was combined with 120 g of soybean powder, 120 g of wheat bran powder, 48 g of yeast powder, and 24 g of casein. This mixture was then combined with the agar solution and heated for an additional 20 to 30 min to ensure uniform mixing and prevent burning. After boiling, 2.4 g of sorbic acid was added and thoroughly mixed. Once the mixture cooled to room temperature, 0.24 g cholesterol, 0.24 g inositol, and 9.6 g vitamin C were added and mixed evenly. Finally, 1.2 g choline chloride was incorporated to complete the preparation of the larval diet [18].

### 2.3. Materials and Reagents

MWCNTs-OH and MWCNTs-COOH were the primary nanomaterials used in this study. Both types of MWCNTs were purchased from Jiangsu Xianfeng Nano Material Technology (Nanjing, China), under product codes XFM71 (MWCNTs-OH) and XFM72 (MWCNTs-COOH). According to the manufacturer’s specifications, these nanotubes have a carbon purity exceeding 98% and similar dimensions, with outer diameters of approximately 4–6 nm and lengths in the range of 0.5–2 μm. Additionally, these functionalized MWCNTs exhibit large specific surface areas (>400 m^2^/g) and high electrical conductivities (>100 S/cm).

### 2.4. Preparation of MWCNT Solutions

Based on previous research, we selected concentrations of 0.04, 0.4, and 4 mg/g of MWCNTs-OH and MWCNTs-COOH for our experimental treatments, with distilled water serving as the control [12]. Precise amounts of each type of nanotube were weighed and separately mixed with 100 mL of deionized water in individual beakers. The mixtures underwent sonication (Model: SN-QX-100, 540 W, frequency: 40 ± 2 kHz; SUNNE, Shanghai Shangpu Instrument Equipment Co., Ltd., Shanghai, China) for a total of 30 min, consisting of 5-minute intervals of sonication followed by 2-minute breaks to ensure even dispersion [12,19].

### 2.5. Characterization of MWCNTs

The morphology and microstructure of the MWCNTs were examined using transmission electron microscopy (TEM). A small droplet of each MWCNT suspension (after sonication) was deposited onto a carbon-coated copper TEM grid and allowed to air-dry. Imaging was conducted using a JEOL JEM-2100 transmission electron microscope (JEOL Ltd., Tokyo, Japan) operated at 200 kV in bright-field TEM mode. Multiple fields were examined to verify nanotube dimensions and assess morphological differences between MWCNTs-OH and MWCNTs-COOH. Outer diameters of nanotubes were measured directly from TEM micrographs using the scale bar for calibration.

Additionally, hydrodynamic particle size distributions and zeta potentials of the MWCNTs were measured using a Malvern Zetasizer system (Nano-ZS, Malvern Instruments Ltd., Malvern, UK). For dynamic light scattering (DLS), diluted samples (0.1 mg/mL in deionized water) were prepared to minimize multiple scattering. Measurements were performed at 25 °C with backscatter detection at 173°, and the intensity-weighted size distribution (Z-average diameter and polydispersity index) was obtained by cumulant analysis. For zeta potential analysis, samples were dispersed in 1 mM KCl to ensure constant ionic strength, and measurements were conducted at 25 °C using the Smoluchowski model to convert electrophoretic mobility to zeta potential. Each sample was measured three times, and mean values ± standard deviations were recorded. The instrument was calibrated with a standard electrophoretic mobility solution before use. Zeta potential values were used to infer the relative surface charge and colloidal stability of the functionalized nanotubes.

The surface chemistry of the functionalized nanotubes was verified using Fourier-transform infrared (FTIR) spectroscopy. FTIR spectra were collected on a Nicolet iS10 spectrometer (Thermo Fisher Scientific, Waltham, MA, USA) over the range of 400–4000 cm^−1^ to detect characteristic vibrational bands of the functional groups. This analysis confirmed the successful introduction of hydroxyl (-OH) and carboxyl (-COOH) groups onto the MWCNT surfaces by identifying their specific infrared absorption features.

Thermal stability and the quantity of functional groups on each nanotube sample were evaluated using thermogravimetric analysis (TGA). Approximately 5–10 mg of each MWCNT sample was placed in a platinum TGA pan and heated from 30 °C to 800 °C at a heating rate of 10 °C/min under a continuous flow of high-purity nitrogen (50 mL/min). TGA was performed on a TA Instruments Q50 analyzer (TA Instruments, New Castle, DE, USA), and the weight percentage was recorded as a function of temperature.

Finally, the dispersion stability of the MWCNT suspensions was evaluated by time-resolved sedimentation observation. Homogeneous aqueous suspensions (0.4 mg/mL) of MWCNTs-OH and MWCNTs-COOH were prepared by ultrasonication, as described in Section 2.4, and then transferred into identical transparent containers. The dispersions were visually observed immediately after sonication and at fixed intervals up to 48 hours [20]. Key time points (approximately 10 s, 1 h, 3 h, 17 h, and 48 h after preparation) were recorded by photographing or visually inspecting the samples against a light background. This qualitative approach enabled a comparison of the sedimentation and aggregation rates of each MWCNT type under identical conditions.

### 2.6. Development Time, Reproduction Period, Fecundity, and Longevity

The impacts of MWCNTs on key life table parameters of *S. frugiperda*, including survival rates of immature and adult stages, developmental duration, fecundity (number of eggs per female), and longevity, were assessed following established protocols with minor modifications [18]. Ultrasonicated stock suspensions containing MWCNTs-OH and MWCNTs-COOH were prepared to achieve uniform dispersion. These suspensions were then incorporated into the artificial diet to obtain the desired concentrations of 0.04, 0.4, and 4 mg/g [19]. The control group received a diet supplemented only with distilled water. Newly emerged first-instar larvae were individually transferred into clean 24-well plates and provided with either the experimental or control diet. When larvae reached the third instar, they were transferred into 12-well plates and continued feeding until reaching the fifth instar stage. Fifth instar larvae were subsequently transferred into 6-well plates and allowed to feed until pupation.

After adult emergence, 25 pairs of newly emerged adults (male and female) from each experimental group were placed separately into fine-mesh nylon net cages (10 cm × 10 cm × 10 cm), with moistened filter paper serving as an oviposition substrate. A total of 840 eggs were randomly collected for the life table study: 720 eggs were allocated to the three different concentrations (0.04, 0.4, and 4 mg/g) of each type of nanotube (MWCNTs-OH and MWCNTs-COOH), and 120 eggs were allocated to the control group. Developmental duration (from egg to adult emergence), survival rate, longevity of each stage, and fecundity were monitored daily until the death of all individuals. The preadult duration was calculated as the total developmental time from egg hatching through the larval, prepupal, and pupal stages until adult emergence. Total longevity was recorded as the entire lifespan from egg hatching until adult death [18]. All experiments were conducted under controlled laboratory conditions of 25 ± 1 °C, 65 ± 5% relative humidity, and a photoperiod of 14L:10D.

### 2.7. Life Table Analysis

The raw data on survival, development, and oviposition of all individuals were analyzed according to the age-stage, two-sex life table theory [21,22] using the TWOSEX-MSChart computer program [23]. Key population parameters calculated included age-stage-specific reproductive value (*v_xj_*), age-stage-specific survival rate (*s_xj_*), age-specific survival rate (*l_x_*), age-specific fertility rate (*m_x_*), age-specific maternity (*l_x_m_x_*), age-stage-specific life expectancy (*e_xj_*), net reproductive rate (*R*_0_), intrinsic rate of increase (*r*), mean generation time (*T*), and finite rate of increase (*λ*).

The bootstrap technique was implemented within the TWOSEX-MSChart program (Version 2024.11.13) [23]. This method involved 100,000 bootstrap replications to estimate variances and standard errors of the population parameters. To compare life table parameters between the treatment and control groups, a paired bootstrap test was performed with the significance level set at 5% [24]. The potential population increase was calculated based on life table data for development, survival, and fertility using the TIMING-MSChart program (Version 2024.11.13) [25]. A simulation predicting the population growth or decline in *S. frugiperda* was conducted starting from an initial population of 10 eggs over a fixed period of 60 days. This simulation aimed to forecast the future population size and age-stage structure of the species.

## 3. Results

### 3.1. Characterization of MWCNTs

**Figure 1:**
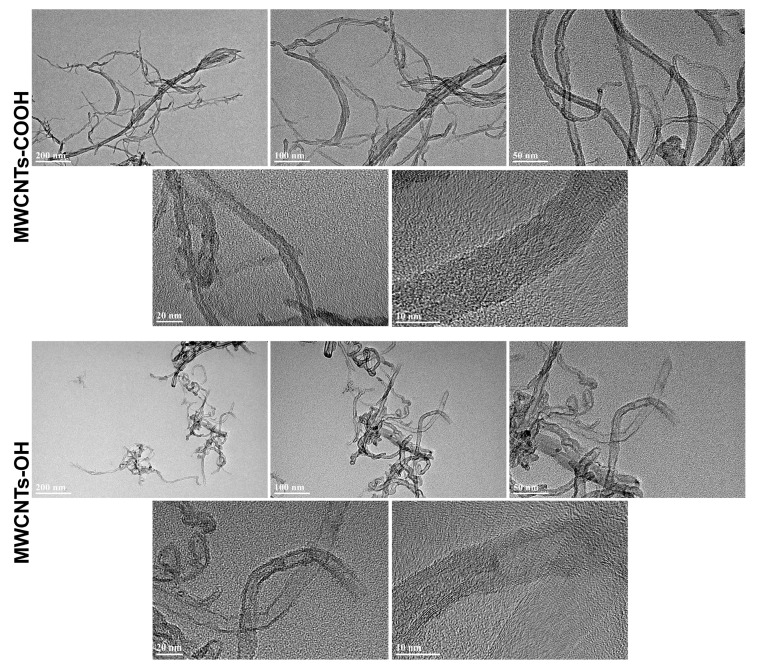
Transmission electron microscopy (TEM) images of carboxylated (-COOH) and hydroxylated (-OH) multi-walled carbon nanotubes (MWCNTs) at different magnifications (scale bars: 200 nm, 100 nm, 50 nm, 20 nm, and 10 nm).

TEM imaging revealed that both functionalized MWCNTs retained the characteristic tubular morphology of multi-walled carbon nanotubes. Representative TEM micrographs (Figure 1) illustrate long, thread-like nanotubes with outer diameters in the nanometer range (approximately 4–6 nm). Internal concentric graphitic layers (walls) were clearly discernible in higher-magnification images, confirming their multi-walled structure. No significant amorphous carbon deposits or residual metal catalyst nanoparticles were observed attached to the nanotubes. Both MWCNTs-COOH and MWCNTs-OH appeared generally similar in length (approximately 1 μm), exhibiting entangled, rope-like aggregates on the TEM grid, likely due to van der Waals interactions. Subtle differences were noted upon close examination: MWCNTs-COOH occasionally exhibited slightly rougher surfaces, more open tube ends, or additional defect sites compared to MWCNTs-OH. This observation suggests that acid oxidative treatment used to introduce -COOH groups created additional sidewall defects or partially shortened the nanotubes, a known effect of harsh carboxylation procedures. In contrast, the MWCNTs-OH surfaces appeared relatively smoother, indicating fewer defects associated with the milder functionalization process. Overall, TEM analysis confirmed that both materials consist of long, thin multi-walled nanotubes without major morphological differences, apart from minor indications of increased defect density in the carboxylated sample (Figure 1).

**Figure 2:**
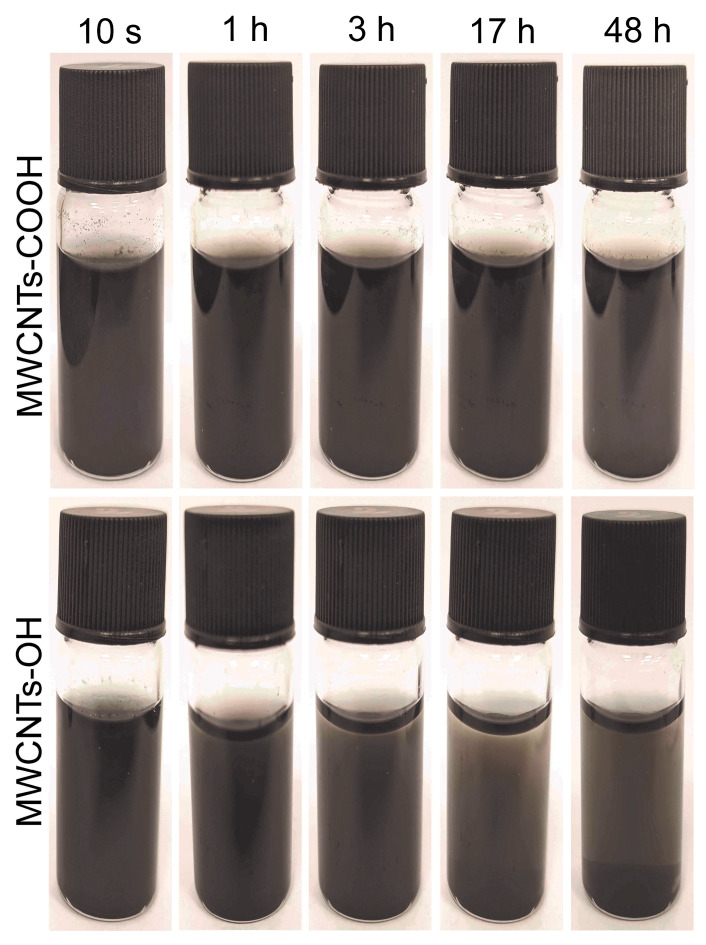
Dispersion stability of carboxylated (-COOH) and hydroxylated (-OH) multi-walled carbon nanotubes (MWCNTs) over various time intervals (10 s, 1 h, 3 h, 17 h, and 48 h).

Marked differences in dispersion behavior were observed between MWCNTs-COOH and MWCNTs-OH when dispersed in water (Figure 2). Although this visual sedimentation method is qualitative rather than quantitative, it provides a clear comparative indication of dispersion stability [26]. Immediately after sonication, both suspensions appeared uniformly black and opaque, without any visible sedimentation. However, dispersion stability differed significantly over time. The MWCNTs-COOH suspension remained largely homogeneous for an extended period: no noticeable settling was observed within the first 12 h, and even after 24 h, only minimal sedimentation was detected at the vial bottom. At 48 h, the MWCNTs-COOH sample showed only a slight gray precipitate, with most nanotubes still suspended, maintaining a dark appearance.

In stark contrast, the MWCNTs-OH dispersion began to lose stability within the first hour. A faint sediment layer appeared at the vial bottom within 1 h, and the sediment gradually increased over subsequent hours. By 6–12 h, a significant portion of MWCNTs-OH had settled, leaving a noticeably clearer supernatant. At the 24-h mark, the MWCNTs-OH dispersion was highly unstable, with most nanotubes aggregated and settled, leaving only a faint tint in the upper liquid. By 48 h, essentially all particles had settled into a compact black sediment layer at the vial bottom.

These observations demonstrate the superior colloidal stability of carboxylated MWCNTs in water compared to hydroxylated MWCNTs. The enhanced stability of MWCNTs-COOH is attributable to their higher surface charge (as demonstrated in zeta potential measurements below), resulting in stronger electrostatic repulsion and increased hydrophilicity due to –COO^−^ groups. In contrast, MWCNTs-OH, with lower surface charge, have a higher tendency to aggregate and precipitate under identical conditions. Visually, after 48 h, MWCNTs-OH formed a compact black pellet at the vial bottom, while MWCNTs-COOH remained predominantly suspended, maintaining a dispersed gray–black appearance (Figure 2). These results highlight the practical advantage of carboxylation in maintaining stable nanotube suspensions in aqueous environments.

**Figure 3:**
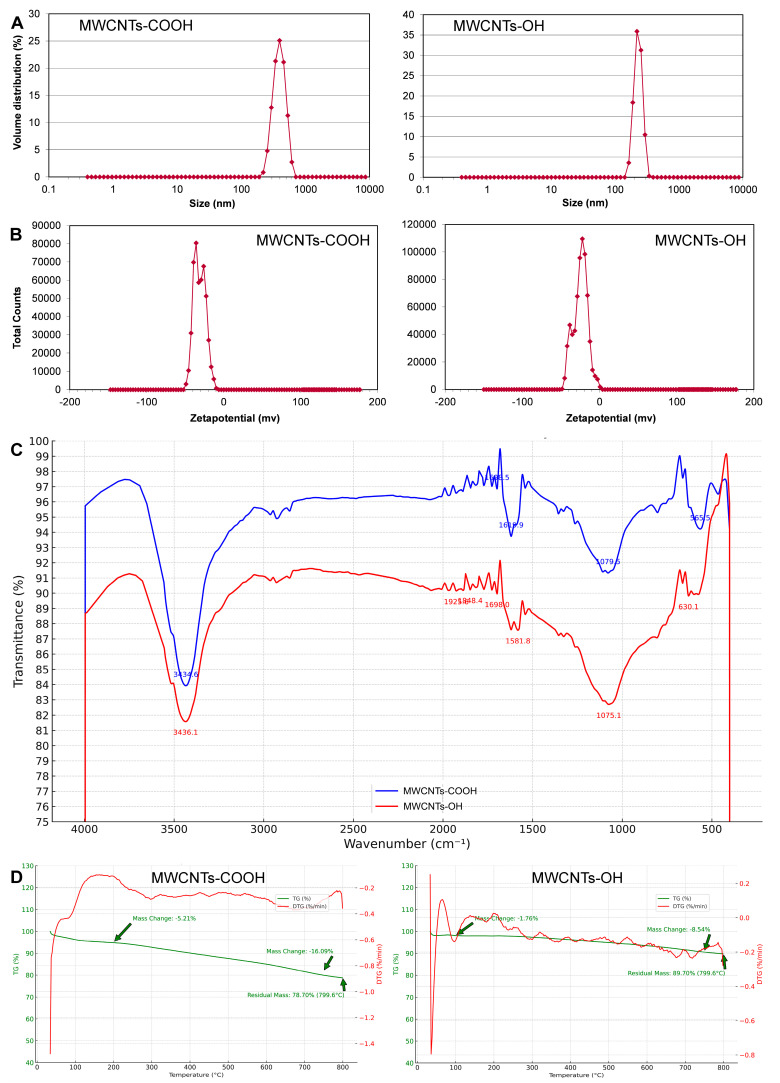
Characterization of carboxylated (-COOH) and hydroxylated (-OH) multi-walled carbon nanotubes (MWCNTs). (**A**) Particle size distribution. (**B**) Zeta potential analysis. (**C**) Fourier-transform infrared (FTIR) spectroscopy. (**D**) Thermogravimetric analysis (TGA).

Dynamic light scattering (DLS) measurements further highlighted differences in dispersion between the two types of nanotubes. The intensity-weighted size distribution of MWCNTs-COOH in water indicated a predominant population of dispersed particles in the submicron range (hundreds of nanometers in hydrodynamic diameter). In contrast, MWCNTs-OH exhibited a broader size distribution skewed toward larger equivalent diameters, frequently in the micron range, suggesting sizable agglomeration. For instance, the Z-average hydrodynamic diameter of MWCNTs-COOH was approximately an order of magnitude smaller than that of MWCNTs-OH (Figure 3A). Although DLS cannot directly measure the actual length of rod-shaped particles, the significantly larger apparent size for MWCNTs-OH indicates extensive clustering into bundles or aggregates in suspension. Conversely, MWCNTs-COOH remained primarily as individual nanotubes or smaller bundles, scattering light as smaller particles. These DLS findings align well with the visual sedimentation tests—carboxylated nanotubes formed finer and more colloidally stable dispersions, whereas hydroxylated nanotubes tended to flocculate (Figure 3A).

Zeta potential analysis quantifies the surface charge of particles in suspension, clearly differentiating the two functionalized MWCNTs (Figure 3B). MWCNTs-COOH exhibited a zeta potential of −30.7 ± 0.2 mV (mean ± SEM), indicating a strong negative surface charge. By comparison, MWCNTs-OH had a zeta potential of −18.1 ± 0.2 mV (mean ± SEM). This difference of over 12 mV in magnitude was statistically significant (*p* < 0.001) and can be attributed to the distinct functional groups: carboxylated nanotubes carry deprotonated –COO^−^ groups, whereas hydroxylated nanotubes contain -OH groups that are less acidic and do not extensively ionize in water (Figure 3B).

FTIR analysis confirmed the successful functionalization of the nanotube surfaces. Both MWCNTs-OH and MWCNTs-COOH displayed a broad O–H stretching band around 3435 cm^−1^ (specifically at 3436.2 cm^−1^ for MWCNTs-OH and 3434.7 cm^−1^ for MWCNTs-COOH), corresponding to hydroxyl groups and possibly adsorbed water on the nanotube surfaces (Figure 3C). Peaks in the 2900–2930 cm^−1^ range, attributed to C–H stretching vibrations of aliphatic groups attached to the carbon framework, were observed in both samples [27]. Importantly, a distinct peak at 1619.9 cm^−1^, attributable to the C=O stretching vibration of carboxylate groups, was observed only in the MWCNTs-COOH spectrum and absent in MWCNTs-OH (Figure 3C) [28]. The presence of the O–H band in both materials and the unique C=O band in the carboxylated nanotubes confirms that hydroxyl and carboxyl functional groups were successfully introduced, clearly differentiating the chemical structures of the two MWCNT types.

Thermogravimetric analysis (TGA) provided insights into the thermal stability and approximate quantity of functional groups present on the nanotube surfaces (Figure 3D). The TGA curves for both MWCNTs-COOH and MWCNTs-OH exhibited an initial slight weight loss at low temperature. Up to approximately 150 °C, MWCNTs-COOH lost around 3–5% of their mass, slightly greater than the 2–3% loss observed for MWCNTs-OH. This early-stage weight loss is attributed to the evaporation of adsorbed water and the release of highly labile functional groups. The greater initial mass loss observed in MWCNTs-COOH aligns with its higher concentration of hydrophilic groups and volatiles from the carboxylation process. Subsequently, a gradual weight loss occurred roughly between 200 °C and 500–550 °C for both samples. In this mid-temperature range, decomposition of covalently bound oxygen-containing functional groups occurred. Notably, MWCNTs-COOH displayed greater mass loss between 200 °C and 400 °C compared to MWCNTs-OH, which have fewer carboxyl groups.

The onset of significant weight loss associated with structural degradation of the nanotube carbon backbone differed slightly between the two samples: MWCNTs-COOH began their primary decomposition at approximately 450–500 °C, whereas MWCNTs-OH resisted weight loss until around 500 °C. The primary mass loss due to combustion or pyrolysis of the carbon framework occurred between 500 °C and 600 °C for both samples, with a peak decomposition rate near 550–580 °C. By 600 °C, most of the nanotube carbon was decomposed under inert atmosphere conditions. Beyond 600 °C, the remaining mass tapered off to a stable residual by 800 °C. Char yield (residual mass at 800 °C under N_2_) was low for both materials: approximately 1–2% for MWCNTs-COOH and around 1% for MWCNTs-OH. In summary, TGA results indicate that MWCNTs-COOH contain a slightly higher fraction of thermally labile oxygen-containing groups (evident by increased low-to-mid temperature weight loss and an earlier decomposition onset), while MWCNTs-OH display slightly greater thermal robustness. Nevertheless, both nanotube types exhibit excellent thermal stability up to several hundred degrees Celsius and minimal inorganic residue, confirming their high purity (Figure 3D).

### 3.2. Impact of MWCNTs on Development, Longevity, and Reproduction of S. frugiperda

The impact of MWCNTs on the development time of *S. frugiperda* was observed across three concentrations (0.04, 0.4, and 4 mg/g), compared to the control (Table 1). Insects reared on the 4 mg/g concentration of MWCNTs exhibited significantly longer egg incubation periods and instar development times (from the first to the sixth instar), except for the egg incubation period in the 4 mg/g of MWCNTs-COOH exposed group. The durations of the prepupal and adult stages did not differ significantly among the three concentrations of the two nanotubes, with only minor exceptions. However, the preadult duration was significantly longer in insects reared on the 4 mg/g concentration of MWCNTs-COOH and MWCNTs-OH compared to those reared on the water control diet (Table 1). These results suggest that MWCNTs can significantly impact the development period of *Spodoptera frugiperda*, particularly at certain concentrations, and emphasize the potential ecological effects of carbon nanotubes on insect life history traits.

The total longevity of female adults was found to be significantly longer when reared on a diet containing a 4 mg/g concentration of both nanotubes compared to the control diet (Table 2). However, there was no significant impact on the total longevity of male adults across the three concentrations of the two nanotubes. Furthermore, the adult preoviposition period (APOP) was notably extended in insects reared on 4 mg/g of MWCNTs-COOH compared to the control diet. On the other hand, no significant changes were observed at the other concentrations. The total preoviposition period (TPOP) was significantly longer in insects fed on the 4 mg/g concentration of MWCNTs-COOH and MWCNTs-OH compared to those on the water control diet. Interestingly, the oviposition period did not show any significant changes between insects exposed to the nanotube concentrations and the control. However, a notable reduction in fecundity was observed in insects exposed to 4 mg/g of MWCNTs-OH compared to the control (Table 2). Overall, the results suggest that MWCNTs have a significant impact on reproductive parameters and longevity in *S. frugiperda*, with effects varying based on the concentration and type of nanotubes used.

### 3.3. Impact of MWCNTs on Population Parameters of S. frugiperda

**Figure 4:**
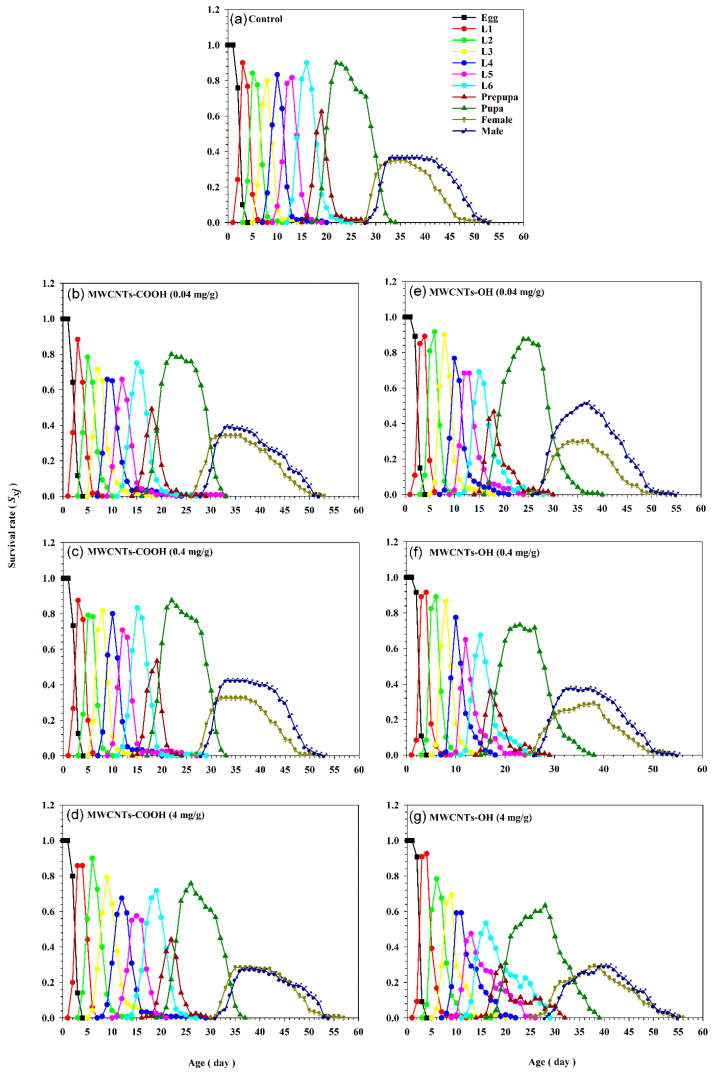
Age-stage-specific survival value (*s_xj_*) of *Spodoptera frugiperda* exposed to three concentrations (0.04, 0.4, and 4 mg/g) of carboxylated (-COOH) and hydroxylated (-OH) multi-walled carbon nanotubes (MWCNTs), in comparison to a control group (water). (**a**) The control group. (**b**) MWCNTs-COOH at 0.04 mg/g. (**c**) MWCNTs-COOH at 0.4 mg/g. (**d**) MWCNTs-COOH at 4 mg/g. (**e**) MWCNTs-OH at 0.04 mg/g. (**f**) MWCNTs-OH at 0.4 mg/g. (**g**) MWCNTs-OH at 4 mg/g.

The survival curve of the *S. frugiperda* cohort, which reflects age-stage survival rates (*s_xj_*), demonstrates extensive overlap between developmental stages due to individual variability in growth rates (Figure 4). This emphasizes the necessity of analyzing stage-specific survival probabilities rather than assuming uniform development within the population [29]. Previous approaches that simplify lifespan division into non-overlapping stages by using averaged developmental timelines—while neglecting inherent variability—risk introducing inaccuracies in survival curve modeling [30]. This study further reveals that increasing concentrations of nanotubes are associated with progressively lower survival rates across all stages compared to control diets. For instance, the sixth instar larvae reared on a control diet had a survival rate of 0.44 by day 18, whereas those exposed to 4 mg/g MWCNTs-OH showed a markedly reduced rate of 0.25 (Figure 4). This underscores the concentration-dependent adverse effects of nanotubes on survival.

**Figure 5:**
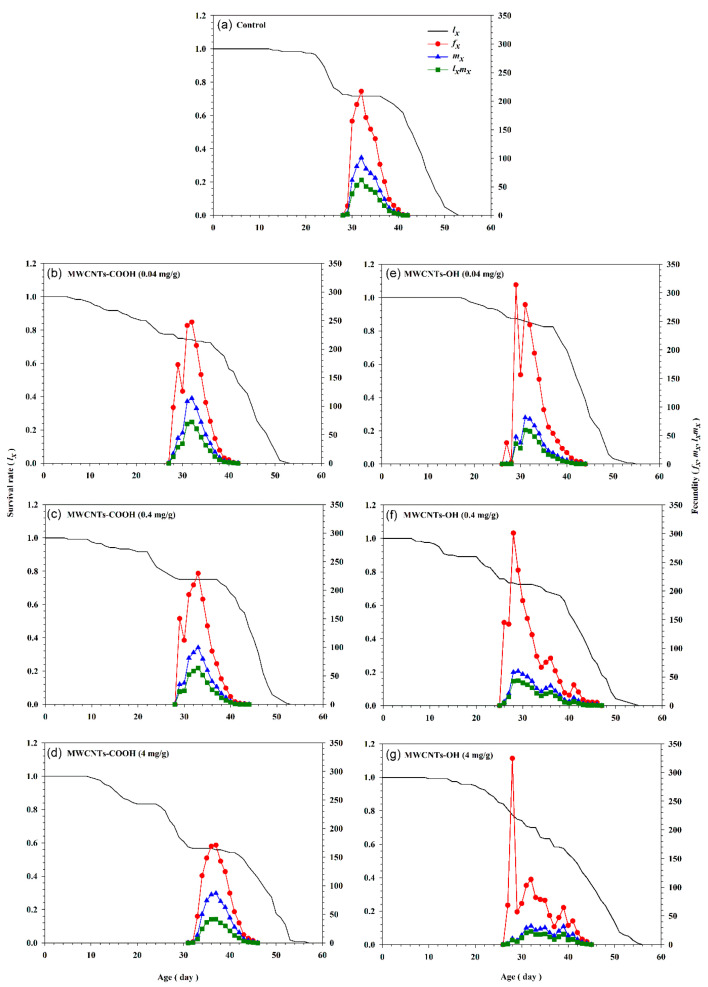
Age-specific survival rate (*l_x_*), age-stage-specific fecundity (*f_xj_*), age-specific fecundity (*m_x_*), and age-specific maternity (*l_x_m_x_*) of *Spodoptera frugiperda* exposed to three concentrations (0.04, 0.4, and 4 mg/g) of carboxylated (-COOH) and hydroxylated (-OH) multi-walled carbon nanotubes (MWCNTs), in comparison to a control group (water). (**a**) The control group. (**b**) MWCNTs-COOH at 0.04 mg/g. (**c**) MWCNTs-COOH at 0.4 mg/g. (**d**) MWCNTs-COOH at 4 mg/g. (**e**) MWCNTs-OH at 0.04 mg/g. (**f**) MWCNTs-OH at 0.4 mg/g. (**g**) MWCNTs-OH at 4 mg/g.

The age-specific survival rate (*l_x_*), age-stage specific fecundity (*f_xj_*), age-specific fecundity (*m_x_*), and age-specific maternity (*l_x_m_x_*) curves for *S. frugiperda* populations reared on varying concentrations of two types of nanotubes are presented in Figure 5. On day 32, insects fed the water control diet had values of 186.10 for *f_xj_*, 86.56 for *m_x_*, and 62.03 offspring for *l_x_m_x_*. In comparison, insects reared on a 4 mg/g of MWCNTs-OH-incorporated diet had significantly lower values of 113.58, 32.45, and 22.72 offspring, respectively. Additionally, on day 22, the average survival rate (*l_x_*) for insects reared on the control diet was 0.97, which decreased to 0.83 for those reared on 4 mg/g of MWCNTs-COOH incorporated diet (Figure 5). These results suggest that higher concentrations of MWCNTs lead to a marked reduction in both fecundity and survival rates in *S. frugiperda*, suggesting a detrimental impact on population dynamics.

**Figure 6:**
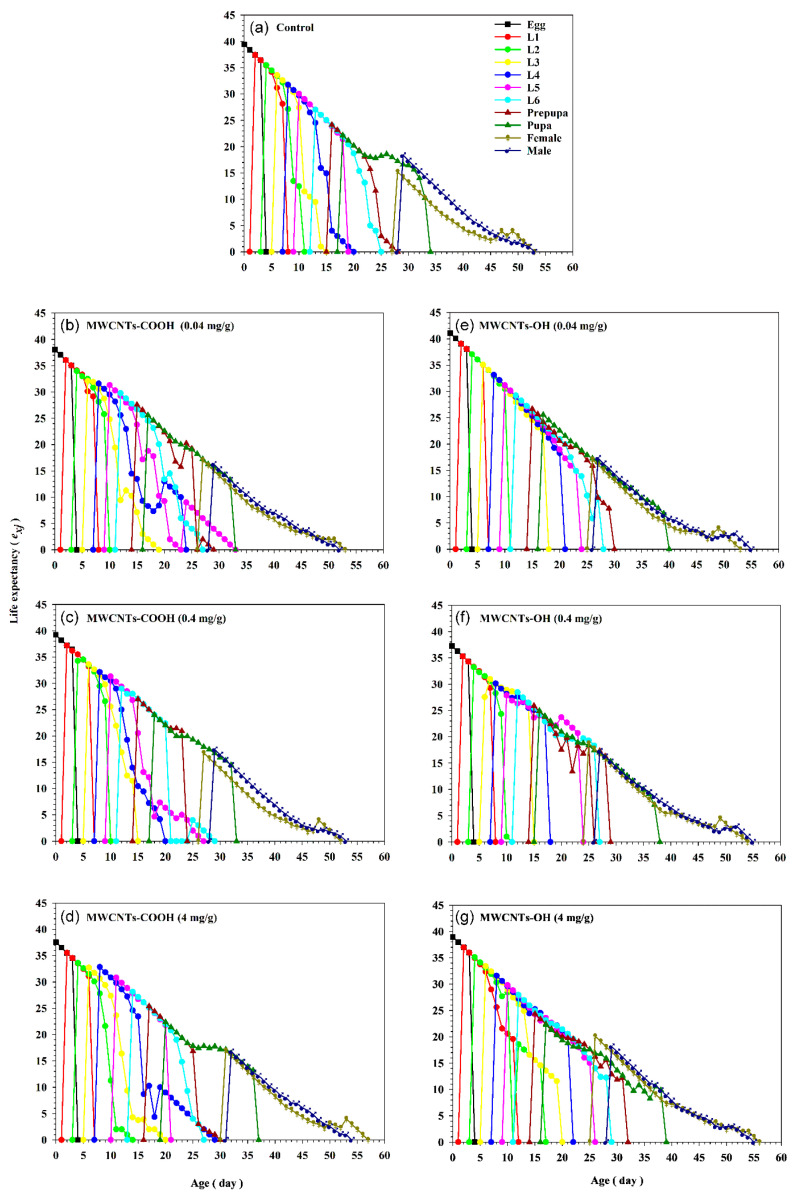
Age-stage-specific life expectancy (*e_xj_*) of *Spodoptera frugiperda* exposed to three concentrations (0.04, 0.4, and 4 mg/g) of carboxylated (-COOH) and hydroxylated (-OH) multi-walled carbon nanotubes (MWCNTs) in comparison to a control group (water). (**a**) The control group. (**b**) MWCNTs-COOH at 0.04 mg/g. (**c**) MWCNTs-COOH at 0.4 mg/g. (**d**) MWCNTs-COOH at 4 mg/g. (**e**) MWCNTs-OH at 0.04 mg/g. (**f**) MWCNTs-OH at 0.4 mg/g. (**g**) MWCNTs-OH at 4 mg/g.

The age-stage specific life expectancy (*e_xj_*), as shown in Figure 6, illustrates the expected lifespan of an individual at a specific age *x* and stage *j* across varying concentrations of two nanotubes and a control group. This analysis demonstrates that *e_xj_* decreases as the concentrations of nanotubes increase (Figure 6). Higher *e_xj_* values for egg and larvae (the first to the fifth instar) were observed when reared on a control diet compared to those insects reared on a diet with 4 mg/g of MWCNTs-OH (Figure 6). The calculation of life expectancy employs the age-stage survival rate (*s_xj_*) without requiring the population to reach a stable age-stage distribution, enabling the prediction of population survival under specific conditions [30]. For instance, on day 15, the life expectancy of the fifth instar larvae was 25 days when reared on the control diet, while it was 24.27 days when reared on a diet with 4 mg/g of MWCNTs-OH (Figure 6). Implementing an age-stage life table allows for the differentiation of individuals of different ages and stages, resulting in a more detailed and precise assessment of life expectancy [30].

**Figure 7:**
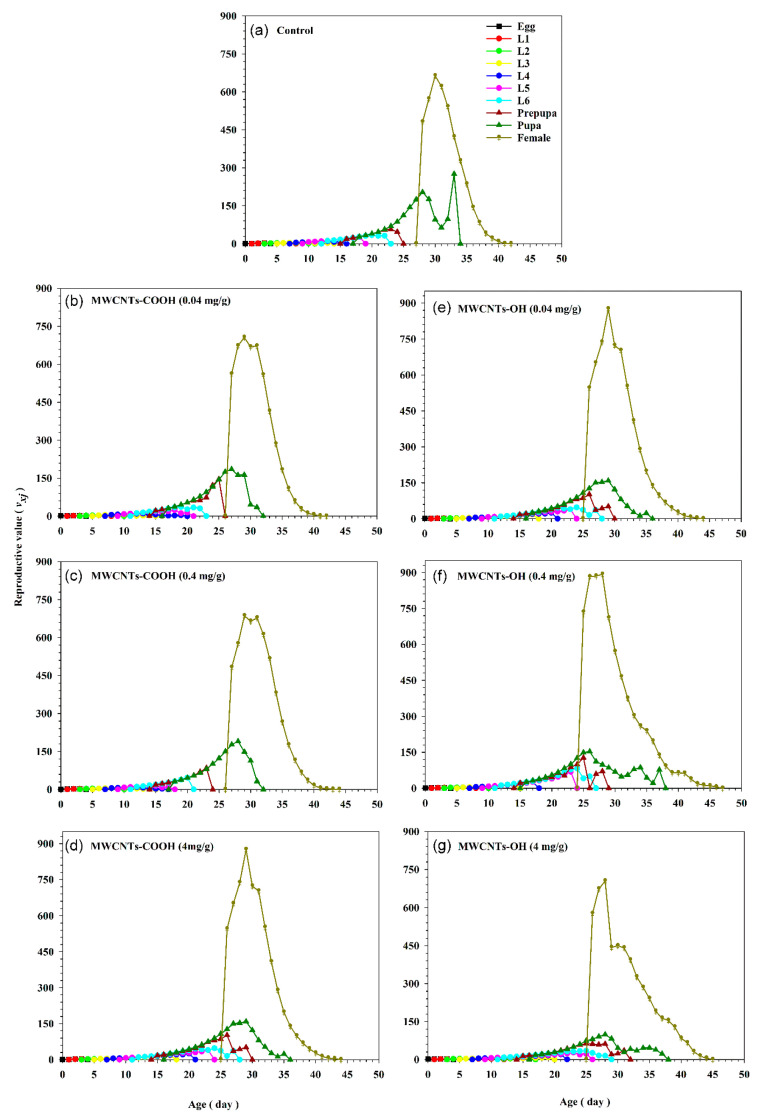
Age-stage-specific reproductive value (*v_xj_*) of *Spodoptera frugiperda* exposed to three concentrations (0.04, 0.4, and 4 mg/g) of carboxylated (-COOH) and hydroxylated (-OH) multi-walled carbon nanotubes (MWCNTs), in comparison to a control group (water). (**a**) The control group. (**b**) MWCNTs-COOH at 0.04 mg/g. (**c**) MWCNTs-COOH at 0.4 mg/g. (**d**) MWCNTs-COOH at 4 mg/g. (**e**) MWCNTs-OH at 0.04 mg/g. (**f**) MWCNTs-OH at 0.4 mg/g. (**g**) MWCNTs-OH at 4 mg/g.

The age-stage reproductive value (*v_xj_*) of *S. frugiperda* quantifies the contribution of an individual of a particular age *x* and stage *j* to the future population (Figure 7). For newborns, the reproductive value (*v*_01_) is equivalent to the finite rate of increase, but significantly increases once the individual begins reproducing. When the insects were reared on a control diet, the *v_xj_* value for 6th instar larvae on day 18 was 27.28 d^−1^, while it was 20.38 d^−1^ for insects reared on a diet with 4 mg/g of MWCNTs-OH. Similarly, female adults on day 30 had a *v_xj_* value of 664.01 d^−1^ with the control diet and 449.62 d^−1^ with the 4 mg/g of MWCNTs-OH diet (Figure 7). Interestingly, as the concentrations of both nanotubes increased, the *v_xj_* values for each stage decreased significantly. This inhibitory effect on moth reproductive values was evident throughout the different stages, from the first instar larvae to adults, when compared to the control group (Figure 7).

### 3.4. Impact of MWCNTs on Life Table Parameters of S. frugiperda

The net reproductive rate (*R*_0_) was significantly lower in insects reared on 4 mg/g of MWCNTs-OH (202.33 ± 31.99 offspring) compared to those reared on the water control diet. However, there were no significant changes in *R*_0_ for other concentrations of both the nanotubes (Table 3). Furthermore, the intrinsic rate of increase (*r*) and finite rate of increase (*λ*) were notably reduced in insects exposed to 4 mg/g of MWCNTs-COOH (0.1469 ± 0.0051 and 1.1582 ± 0.0058 d^−1^, respectively) and MWCNTs-OH (0.1534 ± 0.0058 and 1.1658 ± 0.0068 d^−1^, respectively) compared to those on the water control diet (0.1733 ± 0.0046 and 1.1893 ± 0.0055 d^−1^, respectively). Moreover, the mean generation time (*T*) was significantly prolonged in insects reared on a diet containing 4 mg/g of MWCNTs-COOH (37.87 ± 0.27 days) compared to the control (33.77 ± 0.20 days). Conversely, no significant differences were observed at the other concentrations of both the nanotubes, with a few minor exceptions (Table 3). These results indicate that exposure to higher concentrations of MWCNTs, particularly at 4 mg/g, has a negative impact on crucial life table parameters of *Spodoptera frugiperda*, including reproductive parameters. On the other hand, lower concentrations (0.04 and 0.4 mg/g) have minimal effects on the insects.

### 3.5. Impact of MWCNTs on S. frugiperda Population Projection

**Figure 8:**
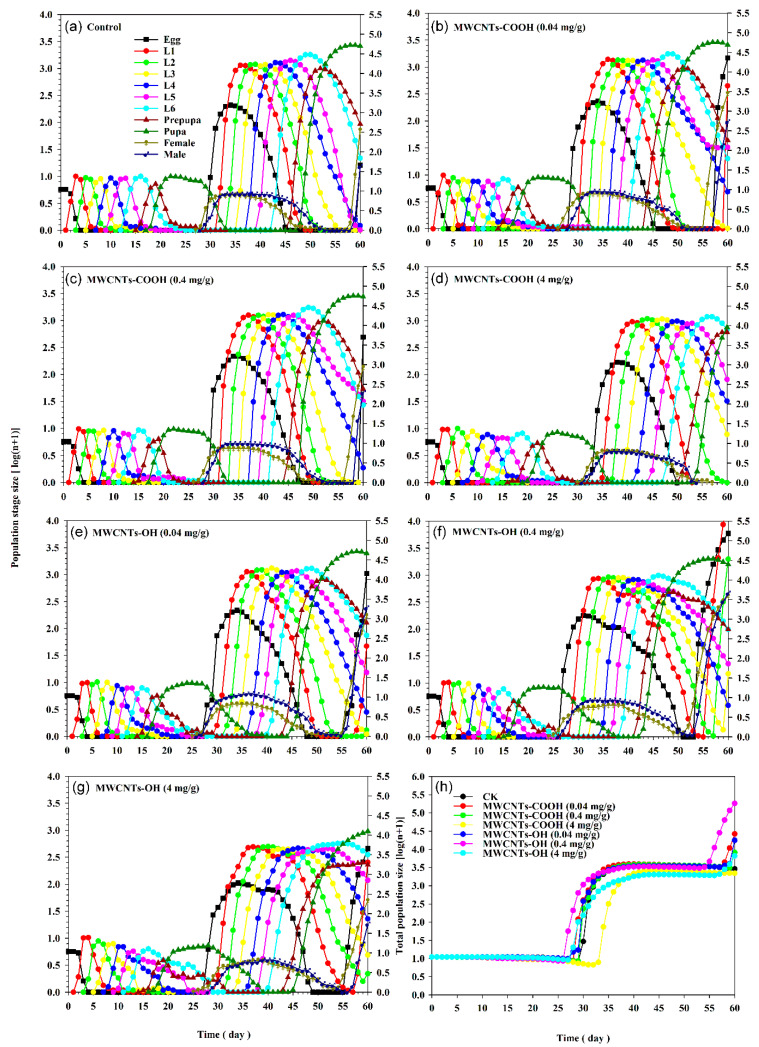
Population projection of *Spodoptera frugiperda* exposed to three concentrations (0.04, 0.4, and 4 mg/g) of carboxylated (-COOH) and hydroxylated (-OH) multi-walled carbon nanotubes (MWCNTs), in comparison to a control group (water). (**a**) The control group showing simulated stage size of the population. (**b**) MWCNTs-COOH at 0.04 mg/g. (**c**) MWCNTs-COOH at 0.4 mg/g. (**d**) MWCNTs-COOH at 4 mg/g. (**e**) MWCNTs-OH at 0.04 mg/g. (**f**) MWCNTs-OH at 0.4 mg/g. (**g**) MWCNTs-OH at 4 mg/g. (**h**) Simulated total population size across all treatments.

The population growth and age-stage structure of *S. frugiperda* at different developmental stages, simulated from an initial population of 10 eggs using the TIMING-MSChart program, are presented in Figure 8. The fastest population growth was observed in the control group in comparison to the insects reared on diets supplemented with nanotubes. As the concentration of nanotubes increased, the stage sizes of the population decreased. After 60 days of simulation, the number of pupae, females, and males in the control group was 2610, 71, and 14, respectively. In contrast, for the population reared on the diet containing 4 mg/g of MWCNTs-OH, the numbers were 954, 48, and 17 for pupae, females, and males, respectively (Figure 8).

## 4. Discussion

The use of nanomaterials in agriculture—especially nano-pesticides—has garnered attention for their potential to improve pesticide efficacy and overall crop productivity. Nano-pesticides can adhere well to plant surfaces and be readily absorbed, which enhances pest control effectiveness [7]. Moreover, nano-carriers such as MWCNTs provide additional benefits: they can improve crop resilience, soil health, and enable the targeted delivery of agrochemicals (including dsRNA for RNA interference applications). These nano-formulations tend to be stable, easy to apply, and highly specific in action, which increases their safety to non-target organisms and allows for seamless integration with conventional pest management methods [31,32].

In this study, two functionalized MWCNTs (MWCNTs-COOH and MWCNTs-OH) were characterized using multiple physicochemical analyses. Transmission electron microscopy (TEM) showed that both materials consist of long multi-walled nanotubes of similar diameter, but the carboxylated MWCNTs (-COOH) exhibited more structural defects than the hydroxylated ones. Carboxylation led to rougher tube sidewalls and more open tube ends, indicating a defect-rich structure [33]. This was corroborated by Fourier-transform infrared spectroscopy (FTIR) and thermogravimetric analysis (TGA): MWCNTs-COOH displayed a higher density of oxygen-containing functional groups. Notably, a distinct C=O stretching band appeared in the FTIR spectrum of MWCNTs-COOH (absent in MWCNTs-OH) [28]. Likewise, TGA showed greater low-temperature weight loss for MWCNTs-COOH, consistent with more labile oxygenated groups and adsorbed moisture on its surface [34]. Together, these findings confirm that the carboxylated nanotubes have a more oxidized surface with numerous defects compared to the hydroxylated nanotubes.

Surface charge measurements and colloidal stability tests revealed further differences between the two nanotube types. MWCNTs-COOH had a strongly negative zeta potential (around −30 mV), reflecting the abundance of deprotonated carboxylate groups on its surface, whereas MWCNTs-OH had a weaker negative charge (approximately −18 mV) due to having fewer polar groups [12]. Consequently, MWCNTs-COOH remained well-dispersed as sub-micron particles in water for extended periods (>24 h) because the strong negative charges caused electrostatic repulsion that prevented aggregation. In contrast, MWCNTs-OHs tended to quickly clump together into larger, micron-sized particles and precipitate within a few hours (1–6 h), owing to their limited electrostatic stabilization. These stark differences in dispersibility and stability likely underlie some of the biological differences observed between the two nanotube treatments. For applications that demand high dispersion stability and strong electrostatic interactions (such as controlled-release formulations), the more heavily functionalized MWCNTs-COOH would be preferable. On the other hand, MWCNTs-OH, with its moderate surface charge and faster aggregation, might be adequate in contexts where only short-term or moderate dispersion is needed.

The ways in which MWCNTs affect insect herbivores are probably a combination of physical and biochemical mechanisms. One likely mode of action is mechanical: when ingested, nanotubes could cause abrasion or irritation to the gut lining, or even block portions of the gut, thereby impairing digestion and nutrient absorption. Additionally, many carbon-based nanomaterials can generate reactive oxygen species (ROS) and induce oxidative stress in biological tissues [35,36]. The surface chemistry of the nanotubes plays a crucial role in these interactions. Carboxylated MWCNTs, with their higher polarity and negative charge, will interact with biological membranes differently than the less polar hydroxylated MWCNTs. Indeed, studies in mammalian systems have reported that -COOH-functionalized MWCNTs can cause more DNA damage compared to pristine or -OH-functionalized nanotubes [37,38]. Both -COOH and -OH surface groups improve dispersibility in aqueous environments and can enhance cellular uptake of nanotubes, potentially increasing their biological activity [36].

In our experiments, the carboxylated nanotubes (MWCNTs-COOH) might have stayed well-dispersed in the insects’ gut fluids and likely passed through the digestive tract with minimal adhesion to gut tissues. This could be due to electrostatic repulsion between the negatively charged nanotube surfaces and the negatively charged cell membranes lining the gut [39,40]. By not sticking extensively to the gut wall, MWCNTs-COOH may cause relatively limited mechanical damage or local disturbance. In contrast, the MWCNTs-OH, which carry a weaker surface charge, probably formed aggregates in the gut and adhered more readily to the gut lining. Such aggregation and adhesion can lead to localized physical damage (abrasion of the epithelium) or create barriers that hinder nutrient absorption. This difference in behavior could explain why we observed more pronounced negative effects on the insects’ development and reproduction with MWCNTs-OH compared to MWCNTs-COOH. Nanotube-induced nutritional stress has been documented in other insect studies as well; for example, carbon nanoparticles disrupted the levels of storage proteins and vitellogenin (an egg-yolk precursor) in caterpillars, contributing to impaired growth and reproduction [41]. Overall, our observations suggest that even subtle differences in nanomaterial surface chemistry can influence the severity of their biological effects. Further research (for example, histological examinations of insect tissues and assays for oxidative damage or enzyme activity) will be necessary to pinpoint the exact mechanisms by which these nanomaterials affect insects.

Our results demonstrate that exposure to MWCNTs can significantly slow the development of S. frugiperda, especially at higher concentrations. In particular, at the highest tested dose (4 mg of nanotubes per g of diet), we observed clear developmental delays: eggs took longer to hatch, and larval stages (instars) were prolonged relative to the control. These findings suggest that the presence of MWCNTs in the diet disrupts normal growth processes, potentially by the mechanisms discussed above. Correspondingly, we also saw that adult females emerging from larvae reared on the nanotube-containing diets laid fewer eggs, indicating a suppression of reproductive capacity. This pattern of slowed development and reduced reproduction at high nanomaterial doses is consistent with reports on other species. For example, house crickets (Acheta domesticus) showed reduced egg production after chronic exposure to graphene oxide (GO) [42], and fruit flies (*Drosophila melanogaster*) exposed to magnetite (Fe_3_O_4_) nanoparticles exhibited decreased fecundity [43].

Other studies reinforce the evidence that carbon-based nanomaterials can negatively affect insect life histories. In *S. frugiperda* larvae, long-term exposure to oxidized MWCNTs and to GO each led to poorer food utilization efficiency and declines in reproductive output; notably, the highest tested GO concentration significantly reduced both fecundity and fertility in that species [19]. In the Oriental river prawn (*Macrobrachium nipponense*), nano-ZnO and MWCNTs caused dose-dependent reductions in reproductive parameters, with the highest concentrations of ZnO (50 mg/L) and MWCNTs (15–20 mg/L) delaying or even halting reproduction altogether [44]. Similarly, carbon nanoparticle (CNP) exposure in another lepidopteran pest, *S. litura*, resulted in distorted adult morphology, reduced egg production, weight loss, and higher mortality. The nanoparticles in that case interfered with key larval hemolymph storage proteins and impeded vitellogenin synthesis, leading to impaired egg development and slower population growth [41]. Consistent with our findings, a recent study on the green peach aphid (*Myzus persicae*) found that functionalized MWCNTs adversely impacted its development, reproduction, and population growth, with the MWCNTs-OH treatment producing more severe effects than MWCNTs-COOH [12].

Taken together, these findings indicate that nanomaterials like MWCNTs can disrupt nutrient absorption or other physiological processes in diverse organisms, leading to longer developmental times, lower fertility, and altered life history parameters. In our study, the impacts on the *S. frugiperda* population dynamics were clearly reflected in the life table metrics. At 4 mg/g of diet, MWCNTs-OH drastically reduced the *R*_0_, meaning females produced far fewer offspring over their lifespans. Both MWCNTs-OH and MWCNTs-COOH at this high dose also caused declines in the *r* and the *λ* relative to the control, indicating that populations exposed to nanotubes would grow more slowly. Additionally, insects reared on 4 mg/g of MWCNTs-COOH showed a significantly longer *T*, suggesting that their overall life cycle (from egg to offspring production) was extended. These changes point to a substantial deceleration of population growth when high levels of nanotubes are present in the diet.

Similar trends in life table parameters have been documented elsewhere. When S. frugiperda larvae were fed graphene oxide, their *R*_0_, *r*, and *λ* values decreased while *T* increased, in a dose-dependent manner [18]. Likewise, cotton bollworm (Helicoverpa armigera) exposed to nanoscale silver and ZnO experienced drops in *R*_0_, *r*, and *λ* [45], and the bulb mite (*Rhizoglyphus robini*) showed reduced population growth rates under exposure to ZnO, MgO, and TiO_2_ nanoparticles [46]. Our study thus adds to a growing body of evidence that carbon nanomaterials can significantly affect the key demographic parameters of pest populations.

It is important to note that the strongest effects in our experiments were observed only at the highest concentration tested (4 mg nanotubes per g diet, equivalent to 0.4% by weight). In practical terms, a dose of this magnitude might be difficult to implement cost-effectively in agricultural settings. If such a high quantity of MWCNTs is required to achieve meaningful pest suppression, the amount of nanomaterial needed per hectare would be considerable. This raises concerns about both economic feasibility and environmental loading. In other words, using MWCNTs alone as a stand-alone nanopesticide could prove impractical unless the effective dose can be substantially reduced. One possible direction for future research is to combine MWCNTs with conventional insecticides or with biopesticides like dsRNA, using the nanotubes as delivery vehicles. Such synergistic approaches might enhance potency enough to allow for lower doses of nanotubes to be used. Additionally, further optimizing the properties of the nanotubes (for instance, adjusting their size or the density of functional groups) could improve their efficacy against pests and potentially lower the dosage required.

It is also worth recognizing that not all studies have found nanomaterials to be harmful to insect development or reproduction. Some investigations report minimal or even neutral/positive effects, highlighting the complexity of nanomaterial–organism interactions. For example, *Drosophila melanogaster* exposed to certain functionalized single-walled and multi-walled CNTs showed no significant changes in fecundity or egg viability [47]. In another case, graphene oxide exposure actually appeared to promote growth and development in the Asian corn borer (*Ostrinia furnacalis*) [48]. Similarly, other carbon nanoparticles like fullerene C_60_, carbon black, and some CNTs did not negatively affect Drosophila growth in laboratory studies [49].

These disparate outcomes suggest that the biological impact of nanomaterials varies greatly depending on the type of nanomaterial, its functionalization, the species in question, and possibly other environmental factors. Our results clearly show that both types of MWCNTs can negatively affect *S. frugiperda*’s life history traits, with the hydroxylated form causing a more pronounced effect. The stronger impact of MWCNTs-OH relative to MWCNTs-COOH may be linked to differences in how they interact with the insect’s biology (as discussed earlier), but it also underlines that even within the category of carbon nanotubes, small changes in chemistry can lead to different outcomes [50]. Additionally, species-specific traits could play a role. The fall armyworm larvae have chewing mouthparts and actively ingest the nanotube-treated diet, which may result in different exposure and damage patterns compared to insects that feed by piercing–sucking or siphoning fluids [51]. Such differences in feeding behavior might explain why, for instance, an insect like an aphid (piercing–sucking mouthparts) could respond differently to nanotubes than a chewing caterpillar. To fully understand these nuances, further investigation is needed. Future studies should examine how various characteristics of MWCNTs (including length, diameter, and surface functional group density) influence their effects on different pests. It will also be important to test these materials in more realistic scenarios: for example, feeding trials on whole plants (to see if plant tissues or defenses modulate the effects) and eventual field trials to evaluate whether MWCNT-induced effects on pests translate to meaningful crop protection. Moreover, mechanistic studies at the molecular level will help clarify how exactly MWCNTs interfere with insect physiology and how specific chemical modifications alter their mode of action [7]. Addressing these questions will be essential for determining whether and how MWCNTs can be reliably and safely used as tools in pest management.

Another critical consideration is the environmental fate and safety of MWCNTs if they are to be used in agriculture. MWCNTs are extremely stable chemically and are not readily biodegradable, meaning they can persist in soil or water for long periods [52]. Studies have shown that carbon nanotubes applied to plants can translocate into plant tissues and even reach edible parts of crops, raising the possibility that these nanoparticles could enter the food chain [10]. Furthermore, high concentrations of CNTs in soil have been reported to affect soil microbiota and fauna; for example, some experiments noted reduced microbial activity in soils amended with large amounts of MWCNTs [53]. Therefore, alongside efficacy, it is crucial to perform comprehensive environmental risk assessments for CNT-based pest control. Key questions include how long functionalized MWCNTs persist in the environment, whether they accumulate in organisms or biomagnify through food webs, and what effects they might have on non-target species such as pollinators, soil invertebrates, or aquatic life if runoff occurs. Ensuring that these nanomaterials do not cause unintended long-term harm to ecosystems will be essential if they are to be integrated into sustainable pest management strategies.

## 5. Conclusions

This study marks the initial assessment of the impact of MWCNTs on S. frugiperda through age-stage, two-sex life table analysis. We found that both hydroxylated and carboxylated MWCNTs can inhibit the insect’s development and reproduction, with MWCNTs-OH generally exerting a stronger negative effect. These results suggest that appropriately formulated MWCNTs could potentially be incorporated into pest control strategies for this invasive pest, for example, not as stand-alone insecticides, but as nano-carriers or additives to deliver traditional insecticides or gene-silencing molecules more effectively. However, we emphasize that the significant biological impacts were mainly observed at the highest concentration tested, and the safety of these nanomaterials for non-target organisms remains uncertain. Therefore, while our work expands the understanding of nanomaterial–pest interactions and points to a novel avenue for pest management, any real-world application of MWCNTs will require further research. Efforts should focus on achieving efficacy at field-realistic (lower) doses and on thoroughly evaluating environmental safety and sustainability. Only with such additional studies can the potential of MWCNT-assisted pest control be harnessed responsibly and effectively.

## Figures and Tables

**Table 1:** Effects of different concentrations of carboxylated (-COOH) and hydroxylated (-OH) multi-walled carbon nanotubes (MWCNTs) on the developmental periods and longevity of *Spodoptera frugiperda*.

Parameters	N	Control	MWCNTs-COOH (mg/g)						MWCNTs-OH (mg/g)					
			N	0.04	N	0.4	N	4	N	0.04	N	0.4	N	4
Egg (d)	120	2.86 ± 0.05 bc	120	2.76 ± 0.06 c	120	2.86 ± 0.06 bc	120	2.94 ± 0.05 ab	120	3.04 ± 0.05 a	120	3.03 ± 0.04 a	120	3.00 ± 0.04 a
1st instar (d)	120	2.09 ± 0.03 b	120	2.13 ± 0.04 b	120	2.13 ± 0.03 b	120	2.42 ± 0.05 a	120	2.06 ± 0.02 b	120	2.13 ± 0.04 b	120	2.56 ± 0.09 a
2nd instar (d)	120	2.23 ± 0.04 bcd	119	2.18 ± 0.04 cd	119	2.16 ± 0.03 d	116	2.84 ± 0.06 a	120	2.33 ± 0.05 b	118	2.29 ± 0.04 bc	119	2.68 ± 0.06 a
3rd instar (d)	119	2.19 ± 0.04 e	115	2.29 ± 0.06 de	117	2.32 ± 0.05 de	106	2.98 ± 0.08 a	120	2.66 ± 0.10 b	116	2.42 ± 0.06 cd	118	2.54 ± 0.07 bc
4th instar (d)	117	2.46 ± 0.05 b	108	2.48 ± 0.08 b	113	2.48 ± 0.08 b	100	3.10 ± 0.06 a	120	2.38 ± 0.08 b	115	2.43 ± 0.07 b	116	2.88 ± 0.10 a
5th instar (d)	117	2.82 ± 0.05 b	104	2.62 ± 0.07 c	109	2.44 ± 0.05 d	99	3.02 ± 0.07 a	120	2.68 ± 0.09 bc	107	2.37 ± 0.07 d	113	3.19 ± 0.13 a
6th instar (d)	117	3.91 ± 0.07 b	101	3.75 ± 0.07 bc	107	3.73 ± 0.05 c	99	4.31 ± 0.05 a	119	3.15 ± 0.05 e	107	3.47 ± 0.06 d	111	4.47 ± 0.10 a
Prepupa (d)	115	2.04 ± 0.05 a	99	2.02 ± 0.05 ab	107	2.05 ± 0.05 a	96	2.13 ± 0.06 a	111	1.96 ± 0.06 ab	102	1.60 ± 0.05 c	108	1.92 ± 0.04 b
Pupa (d)	86	10.30 ± 0.09 bc	88	10.44 ± 0.11 ab	90	10.59 ± 0.11 a	68	10.72 ± 0.11 a	102	10.42 ± 0.08 b	87	10.17 ± 0.10 bc	72	10.14 ± 0.10 c
Preadult (d)	86	30.58 ± 0.14 c	88	29.98 ± 0.16 d	90	30.31± 0.14 cd	68	33.88 ± 0.17 a	102	30.43 ± 0.24 cd	87	29.92 ± 0.30 d	72	32.22 ± 0.38 b
Adult (d)	86	14.77 ± 0.37 a	88	14.76 ± 0.43 a	90	14.98 ± 0.31 a	68	14.51 ± 0.46 ab	102	13.53 ± 0.35 b	87	14.24 ± 0.38 ab	72	14.49 ± 0.47 ab
Total longevity (d)	120	39.43 ± 0.92 ab	120	38.06 ± 1.12 b	120	39.22 ± 1.04 ab	120	37.53 ± 1.23 b	120	41.08 ± 0.73 a	120	37.28 ± 1.13 b	120	38.98 ± 0.99 ab
Female:Male	42:44		41:47		39:51		34:34		37:65		36:51		35:37	

Data are presented as means with standard errors (SE) estimated using 100,000 bootstrap resampling. Statistical differences are indicated by different lower-case letters within each row, which denote significant differences according to a paired bootstrap test at the 5% level of significance.

**Table 2:** Reproductive parameters and longevity of male and female *Spodoptera frugiperda* reared on different concentrations of carboxylated (-COOH) and hydroxylated (-OH) multi-walled carbon nanotubes (MWCNTs).

Population Parameters	N	Control	MWCNTs-COOH (mg/g)						MWCNTs-OH (mg/g)					
			N	0.04	N	0.4	N	4	N	0.04	N	0.4	N	4
Female total longevity (d)	42	43.24 ± 0.51 b	41	44.07 ± 0.64 b	39	43.77 ± 0.51 b	34	47.97 ± 0.59 a	37	43.08 ± 0.60 b	36	43.81 ± 0.69 b	35	46.11 ± 0.78 a
Male total longevity (d)	44	47.36 ± 0.44 ab	47	45.32 ± 0.65 bc	51	46.45 ± 0.43 b	34	48.82 ± 0.77 a	65	44.46 ± 0.54 c	51	44.41 ± 0.70 c	37	47.27 ± 0.73 ab
APOP (d)	42	1.52 ± 0.15 bc	41	1.32 ± 0.09 cd	39	1.56 ± 0.14 bc	34	2.21 ± 0.26 a	37	1.54 ± 0.17 bc	36	1.14 ± 0.07 d	35	1.80 ± 0.17 ab
TPOP (d)	42	31.45 ± 0.25 c	41	30.32 ± 0.22 d	39	31.08 ± 0.20 c	34	35.44 ± 0.29 a	37	31.51 ± 0.37 c	36	30.61 ± 0.57 cd	35	33.17 ± 0.58 b
Oviposition Period (d)	42	7.00 ± 1.40 a	41	7.00 ± 1.43 a	39	8.00 ± 1.34 a	34	6.00 ± 1.74 a	37	7.00 ± 1.64 a	36	7.00 ± 1.88 a	35	6.00 ± 2.05 a
Fecundity (eggs/female)	42	995.98 ± 74.45 ab	41	1156.10 ± 70.59 ab	39	1168.72 ± 63.42 a	34	918.88 ± 105.50 bc	37	1120.70 ± 67.38 ab	36	1164.81 ± 66.48 a	35	693.71 ± 49.08 c

Data are presented as means with standard errors (SE) estimated using 100,000 bootstrap resampling. Statistical differences are indicated by different lower-case letters within each row, denoting significant differences according to a paired bootstrap test at the 5% level of significance.

**Table 3:** Life table parameters [net reproductive rate (*R*_0_), intrinsic rate of increase (*r*), finite rate of increase (*λ*), and mean generation time (*T*)] of *Spodoptera frugiperda* reared on different concentrations of carboxylated (-COOH) and hydroxylated (-OH) multi-walled carbon nanotubes (MWCNTs).

Life Table Parameters	Control	MWCNTs-COOH (mg/g)			MWCNTs-OH (mg/g)		
		0.04	0.4	4	0.04	0.4	4
Net reproductive rate (*R*_0_)	348.59 ± 50.53 a	395.00 ± 55.54 a	379.83 ± 54.13 a	260.35 ± 48.12 ab	345.55 ± 51.46 a	349.44 ± 52.60 a	202.33 ± 31.99 b
Intrinsic rate of increase (*r*)	0.1733 ± 0.0046 a	0.1811 ± 0.0044 a	0.1760 ± 0.0046 a	0.1469 ± 0.0051 b	0.1760 ± 0.0052 a	0.1828 ± 0.0058 a	0.1534 ± 0.0058 b
Finite rate of increase (*λ*)	1.1893 ± 0.0055 a	1.1986 ± 0.0053 a	1.1925 ± 0.0054 a	1.1582 ± 0.0058 b	1.1925 ± 0.0061 a	1.2006 ± 0.0069 a	1.1658 ± 0.0068 b
Mean generation time (*T*)	33.77 ± 0.20 b	33.01 ± 0.23 c	33.74 ± 0.25 b	37.87 ± 0.27 a	33.20 ± 0.32 bc	32.03 ± 0.52 c	34.62 ± 0.67 b

Data are presented as means with standard errors (SE) estimated using 100,000 bootstrap resampling. Statistical differences are indicated by different lower-case letters within each row, which denote significant differences according to a paired bootstrap test at the 5% level of significance.

## Data Availability

The original contributions presented in this study are included in the article. Further inquiries can be directed to the corresponding authors.
